# Neuromuscular Damage and Repair after Dry Needling in Mice

**DOI:** 10.1155/2013/260806

**Published:** 2013-04-09

**Authors:** Ares Domingo, Orlando Mayoral, Sonia Monterde, Manel M. Santafé

**Affiliations:** ^1^Unit of Histology and Neurobiology, Department of Basic Medical Sciences, Faculty of Medicine and Health Sciences, Rovira i Virgili University, Carrer St. Llorenç No. 21, 43201 Reus, Spain; ^2^Physical Therapy Unit, Hospital Provincial de Toledo, Cerro de San Servando s/n, 45006 Toledo, Spain; ^3^Unit of Physiotherapy, Department of Medicine and Surgery, Faculty of Medicine and Health Sciences, Rovira i Virgili University, Carrer St. Llorenç No. 21, 43201 Reus, Spain

## Abstract

*Objective*. Some dry needling treatments involve repetitive and rapid needle insertions into myofascial trigger points. This type of treatment causes muscle injury and can also damage nerve fibers. The aim of this study is to determine the injury caused by 15 repetitive punctures in the muscle and the intramuscular nerves in healthy mouse muscle and its ulterior regeneration. *Methods*. We repeatedly needled the *levator auris longus* muscle of mice, and then the muscles were processed with immunohistochemistry, methylene blue, and electron microscopy techniques. *Results*. Three hours after the dry needling procedure, the muscle fibers showed some signs of an inflammatory response, which progressed to greater intensity 24 hours after the procedure. Some inflammatory cells could still be seen when the muscle regeneration was almost complete seven days after the treatment. One day after the treatment, some changes in the distribution of receptors could be observed in the denervated postsynaptic component. Reinnervation was complete by the third day after the dry needling procedure. We also saw very fine axonal branches reinnervating all the postsynaptic components and some residual sprouts the same day. *Conclusion*. Repeated dry needling punctures in muscle do not perturb the different stages of muscle regeneration and reinnervation.

## 1. Introduction

Myofascial trigger points (MTrPs) are hyperirritable nodules within taut bands of skeletal muscle responsible for sensory, motor (stiffness, weakness, and restricted range of motion), and autonomic dysfunction [1].

Theories regarding the molecular pathophysiology of MTrPs suggest that they are the result of an abnormal depolarization of motor endplates [2]. These dysfunctional endplates translate the electrical potential to muscle contraction which creates localized sarcomere shortenings that give rise to the palpable nodule of an MTrP. After the nodule formation, a cascade of events leading to the local release of nociceptive substances occurs. Using a microdialysis needles, the following nociceptive and sensitizing substances have been detected: bradykinin, tumor necrosis factor-*α* (TNF-*α*), calcitonin gene-related peptide (CGRP), substance P (SP), interleukins (IL 1*β*, 6, 8, and 12), serotonin, and norepinephrine [3, 4]. These substances cause pain and perpetuate abnormal acetylcholine release [2]. 

One of the therapeutic techniques most commonly employed in the treatment of MTrPs is dry needling (DN) [5]. DN consists of the use of the mechanical stimuli of a needle to either eliminate or inactivate the MTrP. Some DN treatments may involve repetitive and fast needle insertions into the MTrP region [1] often obtaining therapeutic benefits [6]. Since the diameter of the needles commonly employed in this treatment ranges from 160 to 450 *µ*m, in contrast to the 20 to 60 *µ*m diameter of a normal adult myocyte, this type of intervention disrupts muscle fibers, motor endplates, and distal axons. This, in fact, is commonly considered to be one of the possible benefits of the technique, by destroying the dysfunctional motor endplates causing the MTrPs as well as the sarcomere shortening of myocytes related to them [1, 5]. Nevertheless, the effect of this neuromuscular injury on muscle fiber and nerve regeneration has not yet been adequately explored. 

Muscle regeneration after minimal lesion was described many years ago [7, 8]. Firstly, there is an inflammatory reaction that cleans the injured area of necrotic debris. Then, activated satellite cells proliferate and merge to create a myotube. Finally, myotubes synthesize actin and myosin myofilaments that assemble sarcomeres to be joined to those previously existing. Efficient muscle regeneration requires a small lesion area and good muscle irrigation [9]. The timing needed to develop the inflammatory reaction and the complete regeneration is already known. Most experimental minimal and mechanical injuries are regenerated within 7 to 10 days [7, 8]. Since neuromuscular synapses seem to be related to MTrP pathophysiology, we hypothesized that the DN can also damage the nerve fibers innervating them. The changes after denervation and factors favoring nervous reinnervation have been largely referred ([10]; see Bishop, 1982 [11], or Stirling and Stys, 2010 [12], for review).

A common initial question regarding DN is whether the laceration injury caused by repetitive insertions of the needle repairs by creating a scar in myofascial tissues or it results in “ad integrum” regeneration. No study to date has addressed this issue either for muscle, nerve, or neuromuscular junction, and this is the main objective of this study.

## 2. Materials and Methods

### 2.1. Animals

Experiments were performed on the* levator auris longus* (LAL) muscle of adult male Swiss mice (30 to 40 days postnatal; Criffa, Barcelona, Spain). This muscle was chosen because it is a subcutaneous and thin muscle with a well-known intramuscular nerve branching pattern and is easy to handle for histological techniques. Following the study, twenty-three mice were sacrificed by exsanguination under anesthesia. The mice were cared for in accordance with the guidelines of the European Community's Council Directive of 24 November 1986 (86/609/EEC) for the humane treatment of laboratory animals. This study was approved by the Ethics Committee of the Rovira i Virgili University.

### 2.2. Dry Needling

**Figure 1:**
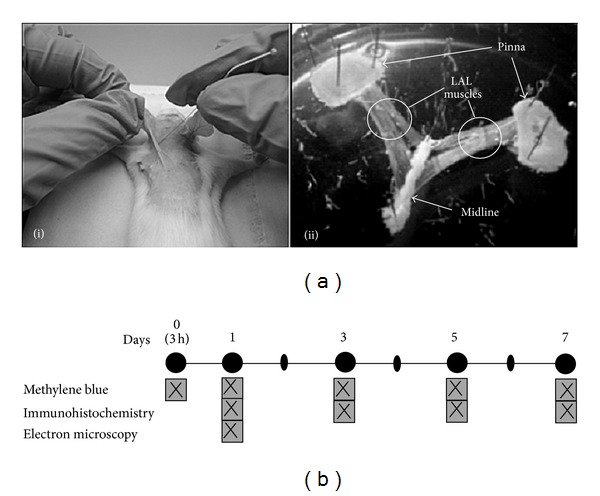
Experimental design and sampling. (a)(i) *Levator auris longus* (LAL) muscle in which repeated punctures are being made with a 0.16 mm thick needle. (a)(ii) Two LAL muscles obtained from the same mouse. (b) Techniques performed and experimental design. After DN, the animals were sacrificed on days one (three hours), two, three, five, and seven. After sacrifice, the muscles were removed, and histological techniques were performed as shown in the figure. In order to minimize the number of animals sacrificed and expenses, the days of extraction and the techniques used were chosen based on a preliminary pilot study.

The animals were anesthetized with 2% tribromoethanol (0.15 mL /10 g body weight, I.P.), and then the treatment was performed (see Figure 1(a)(i)). Fifteen repeated punctures were performed on the muscle with the same type of acupuncture needle commonly used to treat MTrPs. Needles were 0.16 mm thick and 25 mm long. The muscle fiber diameter of rodents and men is similar (for the same level of activity). However, the needle for DN is proportionately greater for the LAL muscle (extremely flat and small, 2 cm^2^) than most human muscles. For this reason, we employed the thinnest needle used for MTrP treatment and few punctures.

To minimize the number of animals sacrificed, both LAL muscles per mouse were used (see the two extracted LAL muscles in Figure 1(a)(ii)). 

### 2.3. Samples

The first sample was obtained three hours after the treatment, and the remaining samples were obtained at one, three, five, and seven days after puncture (see Figure 1(b)). Both LAL muscles were extracted from each animal: the right LAL muscles were processed for immunohistochemistry techniques, and the left LAL muscles were processed for methylene blue staining. The samples processed for electron microscopy were either from the right or the left sides. Muscles were pinned on Silgard in small Petri dishes (see Figure 1(a)(ii)) and then fixed for histological techniques: (a) in 10% neutral formalin for 3 to 10 days for methylene blue; (b) in 4% paraformaldehyde in phosphate buffered saline (PBS, pH 7.4) for 45 minutes at room temperature (~22°C) for immunohistochemistry; (c) in 2 to 5% glutaraldehyde in 0.1 M cacodylate buffer for two hours for transmission electron microscopy.

### 2.4. Immunohistochemistry

Whole LAL muscles were removed and fixed in 4% paraformaldehyde in PBS (pH 7.4) for 45 minutes at room temperature (~22°C). The LALs were double labeled for axons with fluorescein isothiocyanate- (FITC-) conjugated antibodies against 200 kD neurofilament protein (Sigma; 1 : 500 in 1% BSA) and postsynaptic nicotinic acetylcholine receptors with tetramethyl rhodamine isothiocyanate (TRITC)-*α*-BTX. Muscles were mounted in Mowiol with p-phenylenediamine (Sigma). 

### 2.5. Methylene Blue

The samples were exposed to a 1% methylene blue dissolved in 1% borax for two minutes. Subsequently, the samples were washed with distilled water for the three steps of two minutes each. Finally, we proceeded to dehydration and mounting with epoxy resin. 

### 2.6. Electron Microscopy

Tissue samples from LAL muscles containing innervated areas were fixed in a glutaraldehyde (2%) solution for two hours and postfixed in 1% osmium tetroxide for two hours. After dehydration with increasing concentrations of ethanol and acetone, the tissue fragments were embedded in Spurr's resin (plastic) in transverse orientation. Sections 0.5–0.7 nm thick were cut with a Reichert Ultracut E microtome (Leica Microsystems, Bannockburn, IL, USA) and flattened on glass slides by heating on a hot plate. Some sections were stained with methylene blue and mounted with DPX for light microscopic examination.

Ultrathin sections stained with uranyl acetate and lead citrate were observed using a transmission electron microscope at 60 kV (JEOL 1011 Ltd., Tokyo, Japan). Cross-sections of neuromuscular junctions and intramuscular nerves were examined. 

## 3. Results

### 3.1. Muscle Damage

After 15 repeated punctures in the LAL muscle, we analyzed structural changes at several time points. Muscular injuries were quite separated, and we could only observe a single muscle injury by field at low magnification (4x).

**Figure 2:**
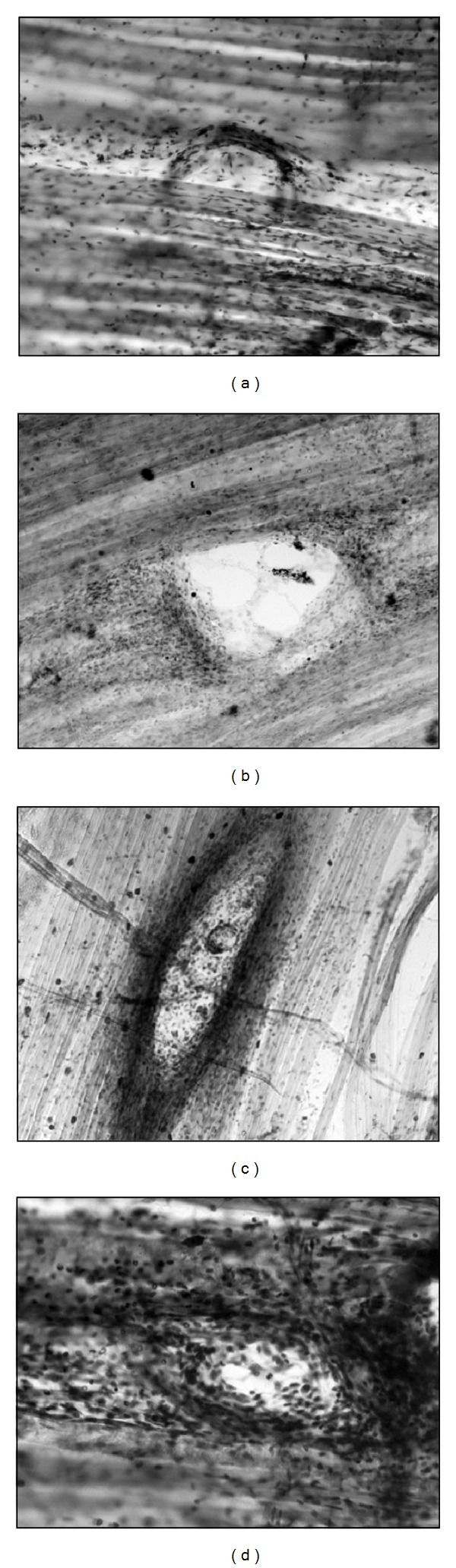
DN injury in muscle fibers. (a) DN injury in connective tissue covering the LAL muscle for the first three hours. Sometimes, the extremely superficial punctures affected only the connective tissue. At three hours, we can see an incipient inflammatory reaction. Initial magnification: 200x. (b) DN injury in the LAL muscle during the first three hours. Incipient inflammatory reaction at the edges of the lesion can be seen. Initial magnification: 100x. (c) The inflammatory reaction is already complete after 24 hours. Initial magnification: 100x. (d) The inflammatory reaction coexists with the initial stages of regeneration after three days. The area without cellularity is smaller than that seen in images (b) and (c). Initial magnification: 400x. The four images have been stained with methylene blue.

**Figure 3:**
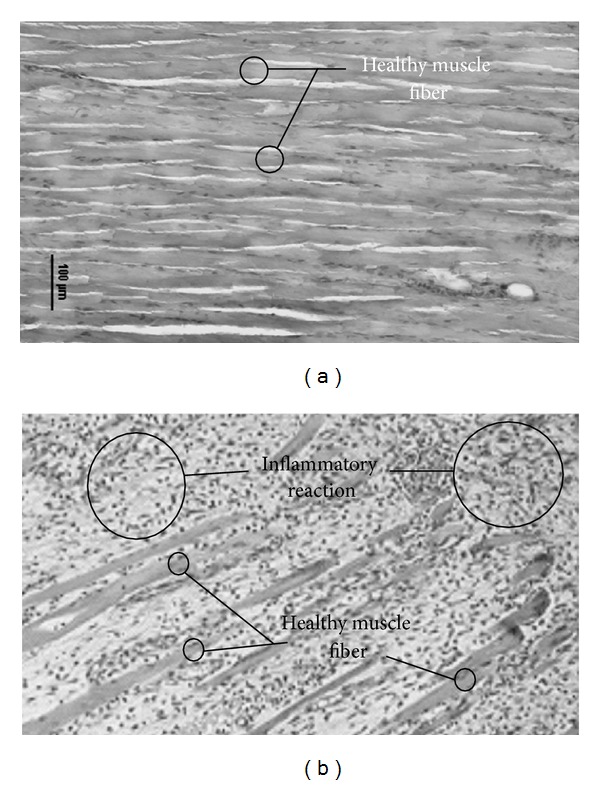
Inflammatory reaction. The figure shows healthy muscle fibers (a) and the inflammatory reaction caused by the needle at the third day after treatment (b). Methylene blue stain. Scale bar: 100 *µ*m.

Three hours after treatment, we found that some punctures were so shallow that they did not reach sufficient depth to injure the muscle fibers and only the connective tissue was injured. The initial inflammatory response was present in the injured connective tissue (Figure 2(a)). As expected, in deeper punctures the damage of muscle fibers was observed on day zero, three hours after intervention (Figure 2(b)). In this situation, the edges of the lesion began to trigger an inflammatory response. At 24 hours after the punctures, a complete inflammatory reaction of the damaged muscle fibers could be observed (Figure 2(c)). Three days after puncture, the muscles were in the initial phase of regeneration presenting an inflammatory reaction (Figure 2(d)). This cleans the necrotic debris of the injured area while preserving the healthy parts of the muscle fibers (compare Figures 3(a) and 3(b)).

**Figure 4:**
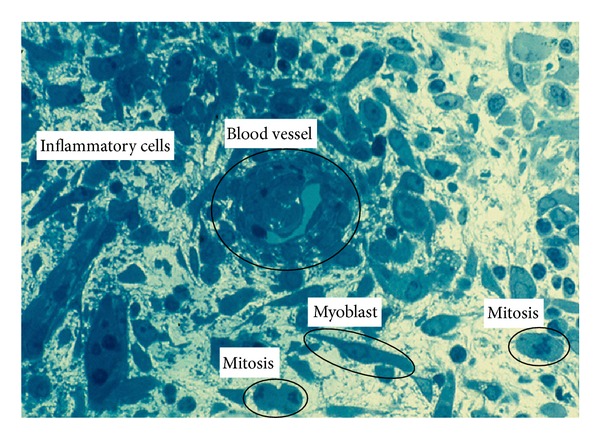
Myoblast proliferation. When the inflammatory cells remove the debris of necrotic muscle fibers, satellite cells are activated and become myoblasts which initiate mitotic proliferation for several hours. Inflammatory reaction, myoblast cells, and mitotic proliferation are coexisting at the same time. Sample was obtained on day three after treatment. Methylene blue stain. Initial magnification: 1000x.

**Figure 5:**
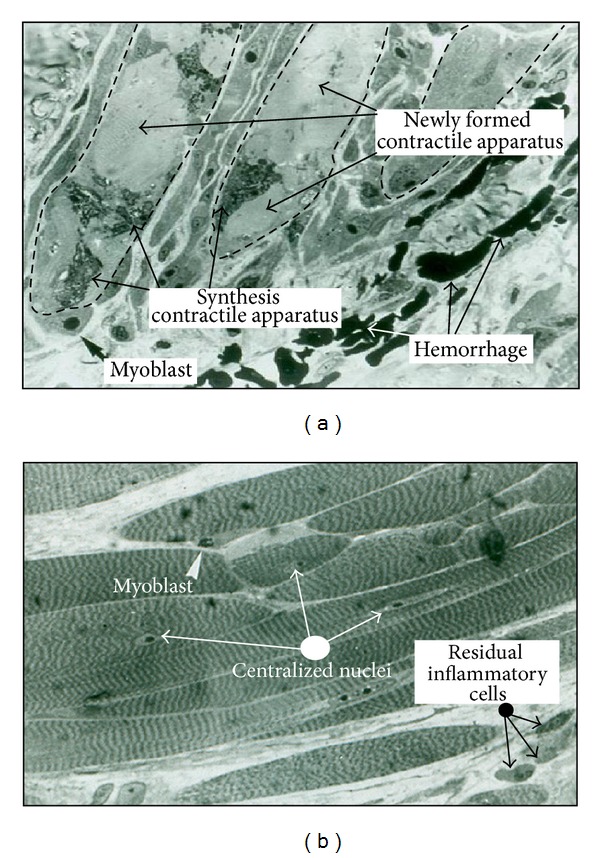
Muscle regeneration. (a) Myotubes. On the fifth day after puncture, myotubes involved in myofibrillar synthesis can be viewed. Note that there are many areas with newly synthesized contractile apparatus. Some myoblasts have not merged to myotube cells and will become satellite cells in the next days. (b) Young muscular fibers. Seven days after puncture, the DN, signs of degeneration and necrosis are less evident than on day five, and some residual inflammatory cells can be seen. As on the fifth day, some myoblasts attached to the young muscle fibers start regressing to satellite cells. Note that the cytoplasm of the muscle fibers is filled by the contractile apparatus. Some myonuclei definitely remain centralized. Methylene blue stain. Sample included in Spurr. Semithin of 1 *µ*m. Initial magnification: 1000x.

On the third day, satellite cells were activated and transformed into myoblasts. These myoblasts initiate a period of mitotic proliferation. The myoblasts are the first step of muscular regeneration.  Figure 4 shows inflammatory reaction cells coexisting with myoblasts. The necrotic debris was completely phagocytized by the inflammatory reaction, and muscle fibers remained healthy at the lesion edges. Between days three and five after injury, the myoblasts fused with each other and with the healthy parts of the injured muscle fibers. The resulting cell of this fusion is the myotube (see Figure 5). Myotubes start the synthesis of actin and myosin (myofibrillogenesis; Figure 5(a)). At day five after injury, the myotubes were already in an advanced stage of myofibrillogenesis, and most of the cytoplasm appeared occupied by the neosynthesized contractile apparatus (Figure 5(a)). Not all myoblasts were fused, and the remaining ones became satellite cells that could be clearly identified by day five and day seven after the treatment (see Figures 5(a) and 5(b)). As observed in humans, mice DN may occasionally induce bleeding (see Figure 5(a)), but this does not seem to interfere with the normal regeneration. 

One week after the treatment, the regeneration was complete. The synthesis had already finished, and most of the cytoplasm was occupied by contractile apparatus (Figure 5(b)). However, occasional nuclei could be observed centrally (Figure 5(b)). It is also a common finding that some mononuclear inflammatory cells remain when muscle regeneration has ended (Figure 5(b)).

In summary, the repetitive mechanical injury in the muscle fiber performed the classical pattern previously described by the original investigators of the muscular regeneration [7, 8].

### 3.2. Nerve Injury

The etiopathogenic explanation of MTrPs involves the neuromuscular synapses [2]. The MTrP is located in the innervation band. Performing DN in skeletal muscles could injure the intramuscular nerves. In our samples, we did not see more than one injury in the same intramuscular nerve branch.

**Figure 6:**
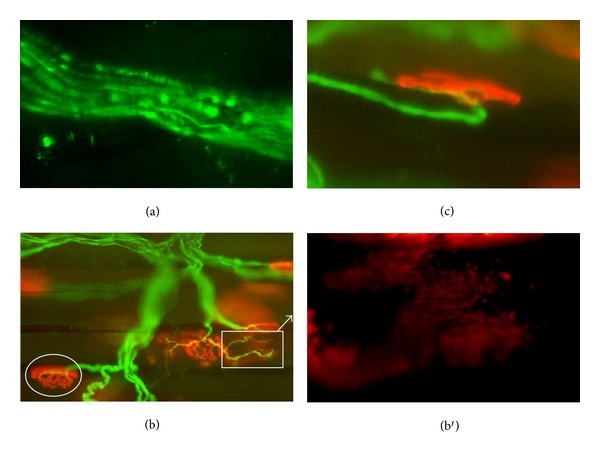
Distal nerve damage by DN. Neurofilament (axon) has been labeled with fluorescein (green) and postsynaptic receptors with *α*-bungarotoxin rhodaminated (red). (a) Intramuscular nerve shows some axons with fragmented neurofilament. Nerve section due to DN is out of the field, and it cannot be observed in this image. Initial magnification: 600x. (b) The circle shows an example of normal endplate: an axon branched covering postsynaptic component perfectly with compact and defined edges. Inside the box, an example of a recently reinnervated endplate is shown: very thin axon and a dispersed postsynaptic component. Initial magnification: 200x. In (b′), this unstructured component is shown in detail. Initial magnification: 600x. (c) Several days after completion of reinnervation, axonal regrowth can still be seen as in this example: a branch finished in axonal growth cone that runs on the postsynaptic component. Initial magnification: 400x.

One day after puncture, we observed fragmented axons with the immunohistochemistry stain (Figure 6(a)). The point of injury was higher than the area shown in Figure 6(a). In few hours, the entire region from the point of injury to the synaptic contacts was fragmented. 

When the fragmentation of axons arrives at the synaptic contact, these synapses will be abandoned. Acetylcholine receptors of the abandoned synapse disperse by the muscle fiber surface. This phenomenon could be observed in our immunohistochemistry images where postsynaptic morphology appeared scattered and unstructured (see Figure 6(b′)). In the third day after puncture, we also found the endplates recently reinnervated by very fine axons covering a spread out postsynaptic component (Figure 6(b), box). During the following days, the receptors would be aggregated below the axon taking the shape of normal adult synapses (see Figure 6(b) circle). At day three after puncture, we could see the axonal growth cone as an axonal dilatation beyond its endplate (Figure 6(c)). This growth cone became residual when the postsynaptic component was reinnervated. For several days, we could see receptors scattered in a process of regrouping under fine axons and axonal growth cones. In both cases, these neuromuscular synapses were functional.

**Figure 7:**
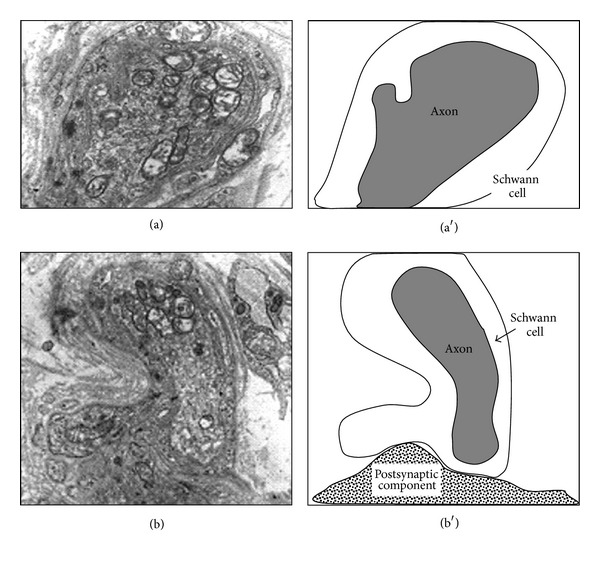
Nerve degeneration and glial participation. Transmission electron microscopy (left) of the first 24 hours after nerve injury accompanied by explanatory diagrams (right). (a) Cross-section of degenerating axon surrounded by a Schwann cell that has lost myelin sheath and is ready for phagocytosis. Initial magnification: 5000x. (b) The terminal Schwann cell completely surrounds the axon terminal and phagocytose. Finally, Schwann cell separates the axon and postsynaptic component. Initial magnification: 5000x.

Electron microscopy results showed that during the first 24 hours after puncture, myelin disappeared and Schwann cells surrounded axon segments to be digested (see Figure 7(a)). Figure 7(b) shows how Schwann cells occupied the synaptic cleft contributing to the nerve terminal degeneration. 

The reinnervation after nerve damage by repetitive mechanical injury was rapid: in three days, neuromuscular synapses were reoccupied. 

## 4. Discussion 

In order to analyze the repetitive mechanical injury in the muscle fiber and intramuscular nerve, we analyzed structural changes after 15 repeated punctures (DN) in the LAL muscle. In this section, we compare the DN injury as described by other authors following periods or phases classically described.

### 4.1. Muscle Damage

Briefly, the usual sequence after muscle injury starts with the inflammatory reaction that removes cellular debris. Then, activated satellite cells, which become myoblasts, initiate a mitotic period. Next, the myoblasts fuse to create myotubes. In the cytoplasm of the myotubes, sarcomeres are synthesized. When the cytoplasm is filled with sarcomeres, the regeneration is complete [7, 8]. We will follow this sequence to discuss our results.

The type of injury studied in this work (DN action) has not been previously reported. However, our results are similar to those described by other authors with other muscle injuries. In this paper, we compare our results with those analyzing minor injuries. Usually, minor damage to muscle triggers a rapid inflammatory response within the first 24 hours after injury (see Figures 2(b) and 2(c)), for example, physical exercise [21], crush plus heat [22], or chemical injury with bupivacaine ([13]; see also Table 1). However, Allbrook [14] described a mild mechanical injury which did not produce the inflammatory reaction until the fifth day. These authors lightly crushed the muscles with forceps applied for two minutes. We believe that the delay observed in the inflammatory reaction is because this was not a pure mechanical injury and vascular involvement also occurred.

Satellite cells typically reside on the surface of healthy adult muscle fibers. Satellite cells are undifferentiated “sleepers” stem cells waiting for the muscular lesions. When the muscle is attacked, satellite cells become myoblasts [7]. As a result of the inflammatory reaction that follows after minimal lesion, the necrotized part of the sarcoplasm becomes a basal lamina cylinder [23, 24]. The first myoblast within this basal lamina cylinder is described usually about 24 hours after the injury, and then a period of mitoses of these stem satellite cells starts [23, 24]. In this sense, our observations of myoblasts coexisting with the inflammatory reaction are similar to results reported by other authors ([23, 24]; see Figure 4). 

 Myoblasts from satellite cells initiate a period of mitosis of 9 to 15 hours. The myoblasts resulting from this mitosis period fuse with each other and with the muscle fibers surrounding the area of injury. The resulting structure is a cell called a myotube [7, 8]. In the literature, most studies on muscle injury from exercise show myotubes occurring in a similar period to that obtained in this study (see Figure 5(a)). For example, myotubes can be seen three days after eccentric running exercise in rats [21] or four days after damage induced by lengthening contractions [25]. 

In the stage of myotubes, actin and myosin are synthesized to create sarcomeres. The synthesis of actin and myosin is called myofibrillogenesis. As described in the literature, the cytoplasm begins to be filled with sarcomeres about four days after exposure to bupivacaine [13] or six days after crush injury [14]. In much more aggressive injuries, such as a complete lesion of the whole muscle, sarcomeres may be observed by day seven [26]. The myofibrillogenesis found in the post-DN treatment is similar in time periods to those obtained with other methods of injury (see Figure 5(a)).

Some myoblasts (9–12%) remain on the surface of myotubes and involute to satellite cells [7, 8]. This is widely reported in the literature (see Péault et al., 2007 [27] for review), and we can see this at five and seven days after the treatment (Figures 5(a) and 5(b)).

When myofibrillogenesis is almost finished, the morphology of the regenerated area is like the normal muscle fiber: whole cytoplasmic volume occupied by sarcomeres and nuclei extruded to the periphery. However, some centralized nuclei can be observed in the regenerated area. This is a common finding after any muscle injury, also after DN (Figure 5(b)). This myonuclei can remain centralized for several years [28]. 

After the first few days of intense inflammatory activity, phagocytic cells progressively disappear. However, in the newly regenerated area, some inflammatory cells persist for some time [29]. We can also see some mononuclear inflammatory cells remaining one week after treatment (Figure 5(b)).

Table 1 shows how the type of injury can modify the regeneration steps at different time points. Indeed, each step observed in this work is consistent with the timing previously described by others [13–16].

In summary, the repetitive mechanical injury in the muscle fiber resembles the classical pattern previously described by other investigators in muscular regeneration [7, 8].

### 4.2. Nerve Injury

As expected, we obtained images of nerve injury with DN. These nerve injuries follow the classic pattern of Wallerian degeneration [10]: initial fragmentation nerve segments, followed by axonal phagocytosis. This phagocytosis at the synaptic level becomes synaptic contact abandoning which produces dispersion of the postsynaptic acetylcholine receptors.

The cascade of events that lead to axonal fragmentation occurs as follows (see Stirling and Stys, 2010 [12], for review): (1) nerve section keeps the distal end without inputs of axoplasmic flow of substances and without axoplasmic transport of organelles; (2) at 24 hours after injury, the distal axonal section already suffers from an energy deprivation; because of a lack of ATP, the ionic pumps (Na^+^/K^+^, Ca^2+^) stop working with a net result of an influx of calcium; finally this calcium activates the calpain protease which degrades the axonal neurofilaments [30, 31], and then fragmented axons can be seen. This phenomenon can be observed in Figure 6(a).

During the first 24 hours after puncture, we can see how Schwann cells surround the axon segments to be digested (see Figure 7(a)). This phenomenon was described previously by Miledi and Slater [32]. During this process, Schwann cells occupy the synaptic cleft contributing to the nerve terminal degeneration as described a long time ago [32, 33] and shown in Figure 7(b). The resulting image of the glial phagocytosis is a synaptic contact abandoned by the axon.

We have obtained an evident dispersion of acetylcholine receptors on the surface of the muscle fibers (see Figure 6(b′)). This phenomenon has already been described by other authors. As described by Thesleff [34], the synaptic component abandoned by degenerated axons cannot maintain aggregation of its postsynaptic receptors. These receptors tend to disperse on the myocyte surface [35, 36]. This situation, called “postdenervation hypersensitivity,” consists of an area larger than the synaptic contact and which is sensitive to acetylcholine molecules [34]. However, in our model the reinnervation occurs faster than previously reported (see Table 2).

Finally, the proximal end of the injured axon elongates following the path of the remaining glia to reoccupy its postsynaptic component [37]. The chemical stimulus favors axoplasmic flow and transport, and a distal dilatation that is responsible for axonal growth, called growth cone, appears [38]. When the axons are broken within the muscle by injuries such as exercise, the nerve-muscle contact is quickly reestablished [39]. In the present study, the nerve injury with DN is made within the muscle, and we find the endplates newly reinnervated at the third day after puncture (see Figure 6(b)). Table 2 shows minor nerve injuries in the bibliography. Faster reinnervation is described in this work by DN.

A growth cone beyond its endplate is a common finding after reinnervation and becomes a residual image for several days [17]. We can see this axonal growth cone at day three after puncture (see Figure 6(c)). 

Axotomy is a complete nerve section which usually occurs with nerve endings separation (see Bishop, 1982 [11], for review). However, DN induces only a partial lesion of the nerve branch without separation of the ends. The sites of experimental nerve injury are usually extramuscular (see, e.g., Verhaagen et al., 1988 [18], or Rich and Lichtman, 1989 [17]), which makes both denervation and reinnervation slower than those described in this study. Table 2 compares several results found in the literature with our own results. 

The nerve damaged by the repetitive mechanical injury is close to the neuromuscular synapse, and its fast reinnervation follows the classic patterns previously described.

## 5. Conclusions

These results show for the first time that dry needling produces intramuscular nerve damage. The lesion produced by skeletal muscle dry needling reproduces the same pattern of muscle injury regeneration already described by other authors, showing that repeated muscle punctures do not interfere with the different stages of muscle regeneration and reinnervation.

The aim of our study was to determine the injury caused by DN in muscle and nerve tissues, and consequently we used healthy tissues. Nevertheless, this could be a limitation to our study, since we do not know whether our results could be extrapolated to the pathological tissue of an MTrP. Future research should replicate these experiments either in the existing animal model for trigger spots [40] or in experimentally induced animal trigger spots [41].

Since the real treatment of MTrPs usually involves more than one treatment, future studies should evaluate whether different treatment regimens would also result in regeneration or would give rise to some degree of fibrosis.

## Figures and Tables

**Table 1:** Some types of muscular injury and degeneration-regeneration times. Some minor muscle injuries were chosen to compare with the DN injury. The degeneration and regeneration periods obtained in this study agree with those previously described for other injury methods. For all items, we considered representative periods of time. For example, for the item “inflammatory reaction,” all publications describe when it starts (indicated by the first number) and the day when abundant inflammatory cells are present (indicated by the second number). Ebbeling and Clarkson, 1989 [15]. Chargé and Rudnicki, 2004 [16]. Benoit and Belt, 1970 [13]. Allbrook, 1962 [14].

Muscle/animal	Gracilis/rat	Tibialis anterior and peroneal/mouse and rabbit	Several muscles/human and rodents	Tibialis anterior/rat	LAL/mouse
	[13]	[14]	[15]	[16]	(this study)
Type of lesion	Local exposure to bupivacaine	Crush injury	Exercise	Local exposure to cardiotoxin	Dry needling
Inflammatory reaction	1st day	3rd-4th days	1st-2nd days	6th hour–4th day	1st–3rd days
Satellite cells proliferation	2nd day	4th–6th days	2nd–4th days	2nd–4th days	3rd day
Myotube	3rd day	6th–10th days	3rd-4th days	6th–10th days	5th day
Young muscular fibers	4th day	15th day	6th–30th days	10th day	7th day

**Table 2:** Minor nerve injuries in the bibliography. We selected studies using nerve injury without separation of the nerve ends, such as crush and neurotoxic. The nerve crush consists of compression with forceps around the nerve for a few seconds. The resultant injury is damaged in all axons without separation of the ends (Lopez-Vales et al., 2008 [19]; Rich and Lichtman, 1989 [17]; Verhaagen et al., 1988 [18]). Similarly, the neurotoxic acrylamide affects all axons without affecting the connective tissue (DeGrandchamp et al., 1990 [20]). Since nerve injury is far from the synaptic contact, some periods are longer than those found in our study. For example, in the report of Lopez-Vales and coworkers [19], the site of sciatic nerve injury was about 45 mm away from the muscle.

Nerve/animal	Nerve of sternomastoid muscle/mice	Nerve of soleus muscle/mice	Sciatic nerve/mice	Sciatic nerve/rat	LAL intramuscular nerve/mice
	[17]	[18]	[19]	[20]	(this study)
Type of lesion	Crush	Crush	Crush	Injection acrylamide	Dry needling
Neurofilament digestion		3th day		4th day	1st day
Phagocytosis of axon	2nd day (area of injury)	6th day (area of injury)	2nd day (area of injury)		2nd day
Reoccupation of postsynaptic component/functional recovery	11th day	8–12 days	21th day	7th day	3th day
Residual growth cones	11th day			7th day	5–7th days
